# Symptom Profiles in Psychotic Disorder Not Otherwise Specified

**DOI:** 10.3389/fpsyt.2020.580444

**Published:** 2020-11-12

**Authors:** Line Widing, Carmen Simonsen, Camilla B. Flaaten, Beathe Haatveit, Ruth Kristine Vik, Kristin F. Wold, Gina Åsbø, Torill Ueland, Ingrid Melle

**Affiliations:** ^1^NORMENT, Division of Mental Health and Addiction, Norwegian Centre for Mental Disorders Research, Oslo University Hospital and University of Oslo, Oslo, Norway; ^2^Early Intervention in Psychosis Advisory Unit for South East Norway, Division of Mental Health and Addiction, Oslo University Hospital, Oslo, Norway; ^3^Department of Psychology, University of Oslo, Oslo, Norway

**Keywords:** DSM, psychosis spectrum, symptom profiles, PNOS, unspecified psychosis

## Abstract

**Introduction:** Approximately 10% of patients with psychotic disorders receive the diagnosis **“**Psychotic disorder not otherwise specified” (PNOS). However, there is a lack of knowledge about the clinical presentations captured by this diagnosis in the mental health services. Therefore, we examined the symptom profiles of participants with PNOS compared to participants with bipolar disorder (BD) and schizophrenia spectrum disorder (SZ) diagnoses.

**Methods:** We here included 1,221 participants from the Thematically Organized Psychosis-study at Oslo University Hospital; 792 with SZ, 283 with BD, and 146 with PNOS, assessed with SCID-I for DSM-IV. The participants with PNOS were categorized into subgroups based on SCID information. The GAF, PANSS, Alcohol Use Disorders Identification Test (AUDIT), and Drug Use Disorders Identification Test (DUDIT) were used to assess function, clinical symptoms, and substance use.

**Results:** In the PNOS group, 44% did not meet the criteria for any specific psychotic disorder, 35.5% had contradictory information making a specific diagnosis difficult, and 20.5% had inadequate information to make a specific diagnosis. The most frequent reason for a PNOS diagnosis was difficulty ruling out a substance-induced psychotic disorder (*n* = 41, 28%). Participants with PNOS were younger and more often first-episode than participants with BD and SZ. They were intermediate between BD and SZ for GAF scores (BD>PNOS>SZ) and PANSS scores (BD<PNOS<SZ) and more often scored above the clinical cut-off for substance misuse as measured by the AUDIT (BD = PNOS<SZ), DUDIT (BD = SZ<PNOS) and for the combination of both these measures.

**Conclusions:** A PNOS diagnosis is more common in first-episode than in multi-episode patients. The diagnosis captures a heterogeneous group of psychotic syndromes, with a severity of symptoms and functional loss that is intermediate between BD and SZ.

## Introduction

Schizophrenia spectrum disorders (SZ) and bipolar spectrum disorders (BD) are commonly seen as two distinct- and separate diagnostic categories. Recent versions of the diagnostic manuals have further divided them into more subgroups, as not all patients with psychotic symptomatology meet the criteria for either SZ or BD at first contact with mental health services. In the fourth edition of DSM (DSM-IV), the diagnosis 298.9 Psychotic disorder not otherwise specified (PNOS) was applied to describe psychotic syndromes that do not fit the description of any of the more specific psychotic disorders, or to cases where there is inadequate or contradictory information on which to base a specific diagnosis (1). As such, the diagnosis PNOS is intended to be temporary and to be reassessed when sufficient information is available. The diagnostic stability of PNOS has also been shown to be low (2), which is consistent with the intended temporary quality of the diagnosis. Existing studies indicate that two-thirds of patients with PNOS will receive a different diagnosis after an average follow-up of 4.5 years, out of which one-third will change to SZ (2). Diagnostic instability, along with shorter duration of illness (3) in patients with PNOS, indicates that the diagnosis is used more frequently in the early stages of psychosis, before the full clinical picture of the disorder has developed. At the same time, about one-third retain the diagnosis of PNOS several years after the onset of illness (2, 4). This may imply, that in addition to these not fully developed features of classical psychotic disorders, PNOS may capture “fully developed” psychotic conditions that simply fail to meet the current diagnostic criteria for SZ or BD.

There are no specific criteria for PNOS, it is a “diagnosis of exclusion” that remains when a patient with clear psychotic symptoms does not meet the criteria for a specific psychotic disorder. DSM-IV, however, offers examples of conditions that will be diagnosed as PNOS. This includes postpartum psychoses, non-bizarre delusions with periods of overlapping mood episodes, auditory hallucinations in the absence of any other psychosis-related features and non-remitted psychotic symptoms lasting less than 1 month, thus not meeting the criteria for brief psychotic disorder (1). Conditions where the information is ambiguous, such as cases of uncertainty between a diagnosis of substance-induced psychotic disorder (SIPD) and a primary psychotic disorder (PPD), are also classified as PNOS. Since alcohol- and drug abuse are common comorbidities in patients with psychotic disorders (5–7), this represent a challenge in diagnostic assessments, particularly in first-episode psychoses (8, 9). The more recent DSM-5 have chosen to include these syndromes in a group called “Other specified schizophrenia spectrum and other psychotic disorder”, which applies to presentations “where symptoms characteristic of a psychotic disorder is present without meeting the full criteria for a specific disorder” (10), in addition to cases meeting the criteria for the attenuated psychosis syndrome (APS). The latter category includes persons with psychosis-like symptoms under the threshold of frank psychosis, considered to be of particular high risk for developing a psychotic disorder (11, 12). DSM-IV PNOS also includes situations where it is difficult to do a complete diagnostic evaluation. This includes situations in which there is not enough information due to time-constraints in emergency room settings or poor report from the patient due to disorganization or memory problems. In the DSM-5 the group defined by insufficient information is categorized as “Unspecified schizophrenia spectrum and other psychotic disorder” (10).

Although PNOS accounts for ~7–12% of patients with first-episode psychosis (3, 13, 14), participants with PNOS are frequently excluded from research studies, which have primarily focused on SZ or BD. There is a real paucity of studies focusing specifically on this group, and the existing studies are limited by small sample sizes (15–17). These studies suggest, however, that patients with PNOS have less severe psychotic symptoms and better short-term outcomes than patients with SZ (16, 17). The findings are supported by studies examining PNOS in youths (18–20). In addition, patients with PNOS appear to have fewer psychotic episodes, shorter durations of illness and better functioning than participants with SZ, according to a larger study comparing all 12 DSM-IV psychotic disorders (3). In the latter, however, no distinction was made between PNOS and brief psychotic disorder. So even though existing literature indicates that patients with PNOS have milder symptoms and better functioning than patients with SZ, there remains a shortage of studies with adequate sample sizes focusing only on adult patients with PNOS. While they are likely to have a better prognosis than patients with SZ, existing studies suggest that PNOS is still a serious condition with significant impairment, even for those who do not convert to SZ (16, 19). This implies that they most often will require comprehensive and long-term treatment. The lack of more specific knowledge will, however, introduce uncertainty concerning treatment planning. Therefore, there is a need to investigate this group in-depth, as increased understanding can contribute to improved interventions.

Finally, while the diagnostic systems are categorical, there are increasing indications that psychotic disorders are dimensional “spectrum” disorders; a group of linked disorders with commonalities both in clinical characteristics and underlying pathologies (21, 22). This necessitates a residual diagnostic category such as PNOS, comprising conditions that lie on the boundaries between, or the margins of, specific categories. In line with this, bipolar disorder not otherwise specified covers conditions with bipolar features that do not meet the criteria for any of the specific BD sub-diagnoses. It is also hypothesized that both BD and SZ are parts of a larger psychosis spectrum (23). Following this notion, it will be of interest to explore the specific symptom profiles captured by the diagnosis of PNOS compared to SZ and BD. To our knowledge, this has not previously been investigated in a large patient sample.

In this study, we will explore the prevalence of different conditions comprised by the DSM-IV PNOS diagnosis, further dividing them into those who fall into the two DSM-5 categories of “Other specified” and “Unspecified” schizophrenia spectrum and other psychotic disorders, respectively, in a large sample of patients with PNOS. We also aim to investigate their symptom severity, functional levels and prevalence of substance misuse comorbidities, compared to participants that meet the diagnostic criteria for BD and SZ, with a focus on differences in symptom profiles between the three diagnostic groups. We hypothesize that the participants with PNOS will be younger, have shorter durations of illness and more often have concurrent substance misuse than participants with BD and SZ, and displaying symptom profiles lying in between BD and SZ.

## Methods

### Subjects

The current study is part of the ongoing Thematically Organized Psychosis Research study (TOP) at the Norwegian Center for Mental Disorders Research (NORMENT), comprising both genetic and clinical research. Participants within the age range of 18 to 65 years meeting a DSM-IV diagnosis of a PPD, that is SZ- and psychotic BD spectrum disorder including PNOS, were recruited from the inpatient and outpatient psychiatric units at the major hospitals in the Oslo area between 2002 and 2018. The hospitals cover a catchment area of 485,000 inhabitants and about 88% of the total population of Oslo. The participants were recruited consecutively by their therapists, and those included in the study during their first treatment were a asked to participate in a long-term prospective longitudinal study. A total of 1,221 participants were included the cross-sectional part of in the study at baseline. Of these, 792 had schizophrenia spectrum disorders; schizophrenia (*n* = 598), schizoaffective disorder (*n* = 141), schizophreniform disorder (*n* = 53), 283 had bipolar disorders; bipolar type I (*n* = 264) and bipolar disorder not otherwise specified (*n* = 19) and 146 had PNOS. Participants who met the criteria for a SIPD, and thus did not have a PPD, were excluded from the study. The same was the case for those with below threshold psychotic symptoms included in the APS. Other exclusion criteria were the presence of pronounced cognitive deficit (IQ below 70), severe brain injury, or not speaking a Scandinavian language.

The study was conducted in accordance with the Helsinki declaration of ethics in medical research and approved by the Regional Committee for Medical Research Ethics and the Norwegian Data Inspectorate. Written informed consent was obtained from all participants prior to the assessments.

### Clinical Assessments

Upon giving informed consent, participants underwent a comprehensive clinical assessment performed by trained physicians or psychologists. The structured assessment took place over several meetings spaced out over several days. DSM-IV diagnosis was established by using the Structured Clinical Interview for DSM-IV Axis I Disorders, modules A-E (SCID-I) (24) supplemented by information from the participants' clinical records as needed. Because of the long-term focus of the associated genetic- and prospective longitudinal studies, the use of DSM-IV was continued through the full study period and not changed to DSM-5 and SCID-5 (25, 26).

A full illness history was also gathered, in addition to information about education, occupation, marital/civil status, and age at illness onset.

#### Classification of Diagnostic Subgroups in Participants With PNOS

The participants with PNOS were classified into subgroups according to the symptomatology that provided the basis of their diagnosis. Based on this, the subgroups were categorized as follows: (1) Psychotic symptomatology that *does not meet the criteria* for any specific diagnosis and (2) Psychotic symptomatology about which there is *contradictory information* to make a specific diagnosis, corresponding to the DSM-5 “Other specified schizophrenia spectrum and other psychotic disorder” diagnosis, and (3) Psychotic symptomatology about which there is *inadequate information* to make a specific diagnosis, corresponding to the DSM-5 “Unspecified schizophrenia spectrum and other psychotic disorder” diagnosis.

#### Measurement of Functional Level and Symptom Severity

All participants were assessed with the Global Assessment of Functioning Scale, split version (GAF) (27). Current positive and negative symptoms were rated using the Structured Clinical Interview for the Positive and Negative Syndrome Scale (SCI-PANSS) (28). We grouped the PANSS items according to the Wallwork five-factor model as studies suggest that this model captures the different symptoms profiles of PANSS better than the original three-factor model (29, 30). Participants with BD were considered to have a history of psychosis if they were either currently psychotic with a score of 4 or higher on PANSS-item P1, P3, P5, P6, or G9, or if they had a previous psychotic episode confirmed by the SCID-I interview.

#### Assessment of Substance Use

All participants were evaluated for DSM-IV substance use disorders (SUD) using SCID-I, Module E. A subsample of 846 participants, also completed Alcohol Use Disorder Identification Test (AUDIT) (31) and Drug Use Disorders Identification Test (DUDIT) (32) to measure the amount and pattern of alcohol and drug use over the past 12 months. Both tests are self-report instruments, AUDIT is used to identify problematic use of alcohol, and DUDIT is used to identify problems with illegal drugs and/or prescription drugs. When assessing AUDIT, a score of 8 for men and 6 for women is usually set as the clinical cut-off for problematic use (33). The clinical cut-off for harmful use was set to 6 for men and 2 for women when assessing drug use by the DUDIT (32).

### Statistical Analyses

The Statistical Package for the Social Sciences (SPSS) for Windows, version 25, was used for statistical analyses. Data were checked for normality, homogeneity of variance and outliers. Group differences were examined with Chi-Square tests for categorical variables and ANOVAs with Bonferroni *post hoc* tests for continuous variables. For continuous variables with unequal variances, Welch's ANOVA and GAMES-Howell *post hoc* tests were used. To illustrate the differences between the groups, we created z-scores for the PANSS ratings for the BD and PNOS patient groups based on the mean and standard deviation of the SZ patient group. All tests were two-tailed with an alpha level of 0.05.

In cases of 1 or 2 missing items on PANSS, AUDIT, or DUDIT, imputations were made by replacing the missing item with the group mean. This was done in 14 cases of PANSS scores replacing item P5 (1 participant), P7 (1), N5 (3), G3 (1), G4 (2), G5 (2), G11 (1), G12 (1), G13 (1), G14 (1), in 16 cases of AUDIT scores, replacing item A1 (1), A2 (4), A3 (4), A6 (1), A7 (3), A8 (2), A9 (1), and 15 cases of DUDIT scores, replacing item D1 (3), D2 (2), D3 (2), D4 (1), D5 (1), D6 (1), D8 (4), D9 (1).

## Results

### Demographics

Demographic and clinical data are presented in Table 1. The participants with PNOS were statistically significantly younger, were more often first-episode and had shorter illness durations than those with SZ and BD. Age at illness onset did not differ significantly between the three groups. Participants with PNOS and SZ had the same length of education, but participants with PNOS were more often in work/education, and they were also more often married/cohabiting compared to participants with SZ. A higher proportion of participants with PNOS were diagnosed with a current SUD compared to participants both with BD and with SZ.

**Table 1:** Demographics and illness course.

	**1. BD (*n* = 283)**	**2. PNOS (*n* = 146)**	**3. SZ (*n* = 792)**	**Anova/Chi-square analysis**				
				**F/χ^2^**	**df**	***p***	**Effect size**	***post hoc***
**Demographics**								
Sex, female, *n* (%)	163 (57.6)	57 (39.0)	326 (41.2)	24.94	2	<0.001	0.143	PNOS, SZ < BD
Age, years, mean (SD)	34.2 (12.3)	28.1 (8.8)	30.7 (9.8)	17.83	2, 354	<0.001	0.027	PNOS < SZ < BD
Married/Live-in partner, *n* (%)	95 (33.6)	34 (23.4)	117 (14.8)	46.86	2	<0.001	0.196	SZ < PNOS < BD
Years in education, mean (SD)	14.5 (2.9)	13.1 (2.8)	12.8 (2.8)	35.39	2, 1209	<0.001	0.058	PNOS, SZ < BD
Working or student, *n* (%)	112 (39.7)	65 (44.5)	149 (18.9)	72.35	2	<0.001	0.244	SZ < PNOS, BD
**Illness course**								
Age at onset, years, mean (SD)	22.7 (8.8)	24.0 (8.4)	23.7 (8.0)	1.7	2	0.183		n.s.
Duration of illness, years, mean (SD)	11.3 (10.2)	4.5 (5.5)	7.1 (7.6)	38.36	2, 361	<0.001	0.060	PNOS < SZ < BD
First-episode, *n* (%)	113 (39.9)	93 (63.7)	333 (42.0)	26.10	2	<0.001	0.146	BD, SZ < PNOS
Substance use disorder, *n* (%)	29 (10.2)	38 (26.0)	124 (15.7)	18.18	2	<0.001	0.122	BD < SZ < PNOS

BD, bipolar disorder; PNOS, psychotic disorder not otherwise specified; SZ, schizophrenia spectrum disorder; n.s, non-significant.

### Diagnostic Subgroups of PNOS

The distribution of participants with PNOS over the predefined subgroups is shown in Table 2. The first subgroup (44% of all PNOS participants) consisted of conditions with psychotic symptomatology that *did not meet the criteria* for any specific psychotic disorder. The most frequent profiles in this subgroup were conditions of persistent non-bizarre delusions with overlapping mood episodes (*n* = 21, 14%), conditions with hallucinations without other psychotic features (*n* = 19, 13%), conditions with SZ symptoms without markedly impaired functioning (*n* = 14, 10%), and conditions with persistent delusions with markedly impaired functioning (*n* = 10, 7%). The second subgroup (35.5% of the PNOS participants) consisted of conditions with psychotic symptomatology about which there was *contradictory information*. In this group the most common presentations were conditions that met Criterion A for either SZ (*n* = 28, 19%) or delusional disorder (*n* = 13, 9%), but where SIPD could not be fully ruled out; most commonly because of ongoing substance use. These two subgroups correspond to the DSM-5 “Other specified schizophrenia spectrum and other psychotic disorders.” The third subgroup (20.5% of the PNOS participants) included conditions with psychotic symptomatology about which there was *inadequate information* to make a specific diagnosis. Participants were classified in this group predominantly because they were unable to provide sufficiently detailed information (*n* = 12, 8%) or if some symptoms were too vague to establish a specific diagnosis with certainty (*n* = 11, 7.5%). This subgroup correspond to the DSM-5 “Unspecified schizophrenia spectrum and other psychotic disorders.”

**Table 2:** Diagnostic subgroups of participants with PNOS.

**1. Psychotic symptomatology that does not meet the criteria for any specific psychotic disorder, *n* (%)**	**64 (44%)**
a. Persistent non-bizarre delusions with periods of overlapping mood episodes that have been present for a substantial portion of the delusional disturbance	21 (14.4%)
b. Hallucinations without other psychotic features	19 (13.0%)
c. Meets Criterion A for Schizophrenia, but functioning is not markedly impaired	14 (9.6%)
d. Meets Criterion A for Delusional Disorder, but functioning is markedly impaired	10 (6.8%)
2. **Psychotic symptomatology about which there is contradictory information**, ***n*** **(%)**	52 (35.5%)
a. Meets Criterion A for Schizophrenia, but Substance-Induced Psychotic Disorder cannot be ruled out	28 (19.2%)
b. Meets Criterion A for Delusional Disorder, but Substance-Induced Psychotic Disorder cannot be ruled out	13 (8.9%)
c. Other cases where there is contradictory information	11 (7.5%)
3. **Psychotic symptomatology about which there is inadequate information to make a specific diagnosis**, ***n*** **(%)**	30 (20.5%)
a. Participant unable to provide sufficiently detailed information about symptomatology to make a specific diagnosis	12 (8.2%)
b. Vague psychotic symptomatology	11 (7.5%)
c. Other cases where information is missing	7 (4.8%)
Total	146 (100%)

PNOS, psychotic disorder not otherwise specified.

### Symptom Profiles

**Figure 1:**
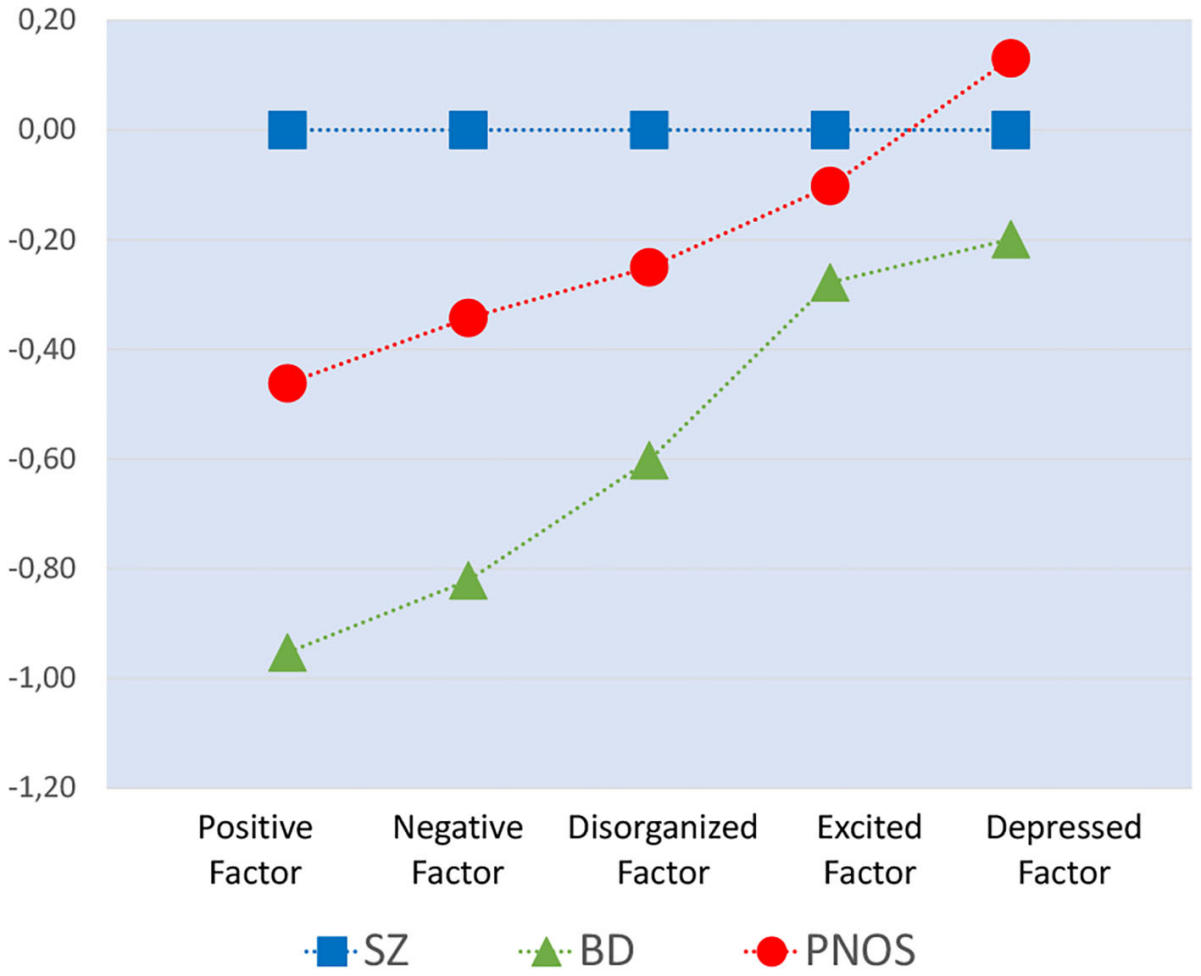
The symptom profiles of the three diagnostic groups, based on z-scores for the five PANSS factors, with the z-scores for the BD-group and PNOS-group relative to the SZ-group. The z-scores are based on the SZ groups' means and standard deviations. The background data for this figure are included in Table 3. Note that the factors represent discrete categories and the dashed lines are used for illustration purposes.

Group comparisons for GAF and PANSS for the three diagnostic groups are presented in Table 3. The participants with PNOS were intermediate between the other two groups for both GAF-S and GAF-F; however with symptom severity (GAF-S) rated closer to the SZ group and functioning (GAF-F) closer to the BD group. The PNOS participants were also intermediate on the Positive, Negative and Disorganized / Concrete Factor of PANSS (BD < PNOS < SZ). For the Depressed Factor, both SZ and PNOS scored higher than BD, while for the Excited Factor, we only found statistically significant differences between BD and SZ (BD < SZ). The symptom profiles of the three diagnostic groups, based on z-scores for the five PANSS factors are shown in Figure 1.

**Table 3:** Clinical characteristics.

	**BD (*n* = 283)**	**PNOS (*n* = 146)**	**SZ (*n* = 792)**	**Anova/Chi-square analysis**				
				**F/χ^2^**	**df**	***p***	**Effect size**	***post hoc***
**PANSS**								
Positive Factor, mean (SD)	6.2 (2.9)	8.3 (2.9)	10.4 (4.4)	159.14	2, 418	<0.001	0.206	BD < PNOS < SZ
Negative Factor, mean (SD)	8.9 (3.8)	11.6 (5.6)	13.6 (5.8)	122.50	2, 372	<0.001	0.166	BD < PNOS < SZ
Disorganized/Concrete Factor, mean (SD)	4.4 (1.7)	5.3 (2.2)	6.0 (2.7)	67.62	2, 393	<0.001	0.099	BD < PNOS < SZ
Excited Factor, mean (SD)	5.2 (1.8)	5.6 (2.0)	5.8 (2.3)	11.16	2, 377	<0.001	0.016	BD < SZ
Depressive Factor, mean (SD)	7.4 (3.1)	8.4 (3.1)	8.0 (3.2)	6.45	2, 1218	0.002	0.011	BD < PNOS, SZ
**GAF**								
Symptoms, mean (SD)	58.0 (12.8)	47.3 (11.1)	42.6 (11.7)	159.8	2, 352	<0.001	0.206	SZ < PNOS < BD
Functioning, mean (SD)	54.9 (13.3)	50.1 (13.0)	43.2 (11.2)	95.8	2, 331	<0.001	0.134	SZ < PNOS < BD
**Substance use (*****n*** **=** **846):**	BD (*n* = 199)	PNOS (*n* = 119)	SZ (*n* = 528)					
AUDIT-score above clinical cut-off, *n* (%)	86 (43.2)	59 (49.6)	180 (34.1)	12.38	2	0.002	0.121	SZ < BD, PNOS
DUDIT-score above the clinical cut-off, *n* (%)	38 (19.1)	54 (45.4)	143 (27.1)	25.98	2	<0.001	0.175	BD < SZ < PNOS
Both AUDIT and DUDIT-score above clinical cut-off, *n* (%)	30 (15.1)	38 (31.9)	82 (15.5)	19.20	2	<0.001	0.151	BD,SZ < PNOS

BD, bipolar disorder; PNOS, psychotic disorder not otherwise specified; SZ, schizophrenia spectrum disorder; GAF, Global Assessment of functioning; PANSS, Positive and Negative Syndrome Scale; AUDIT, Alcohol Use Disorder Identification Test; DUDIT, Drug Use Disorders Identification Test.

### Substance Use

Participants with PNOS and BD scored statistically significantly more often over the clinical cut-off of AUDIT than participants with SZ. Participants with PNOS scored statistically significantly more often over the clinical cut-off of DUDIT than participants with SZ and BD. A total of 32% the participants with PNOS had scores above clinical cut-offs for both AUDIT and DUDIT compared to 15% of participants with BD and 15.5% of participants with SZ (*p* < 0.001). However, when excluding those who were assigned a PNOS diagnosis because SIPD could not be ruled out, the remaining PNOS participants did not have a higher incidence of SUD or more frequently a score above the clinical cut-off for AUDIT or DUDIT than the two other groups.

## Discussion

We here examined a large sample of participants diagnosed with PNOS. They were found to be younger, more often in their first illness episode and had higher rates of substance use than the participants diagnosed with SZ or BD. The largest sub-group of the PNOS cases were diagnosed as such because a SIPD could not be fully ruled out due to ongoing substance use. There was no overrepresentation of substance use in the remaining PNOS sample when these cases were excluded from the analyses.

Findings of similar ages of onset, but shorter durations of illness in the PNOS group than in the SZ group are in line previous studies (3, 16). The short observation time and large proportion of first treatment is consistent with the notion that conditions classified as PNOS may be early manifestations of what will later become more specific psychotic disorders, supported by studies showing that a high proportion of participants with PNOS converts to other diagnoses at a later stage (2, 4, 34). Our finding that PNOS participants were younger than the other two groups is not seen in previous studies (3, 16). This is probably due to the fact that more of the PNOS participants were in their first illness episode, than the participants with SZ and BD in the current study.

Our large sample of participants with PNOS allowed us to explore the types of conditions that were assigned this diagnosis by SCID for DSM-IV. A substantial proportion of participants with PNOS was given this diagnosis due to the inability to fully ruling out the possibility of a SIPD in patients with ongoing substance use. Since many patients with a PPD have high levels of substance use (5–7), this differentiation is difficult, especially in first episodes where there are not sufficiently long periods of abstinence to evaluate what happens if the substance use stops (35). The differentiation is made particularly challenging since studies do not find any consistent differences in psychopathology between individuals with SIPD and PPD (9). In addition, about 25% of the patients initially diagnosed with SIPD at first contact will receive a SZ diagnosis at subsequent follow-ups, thus adding to the diagnostic difficulties (36).

Our finding of high AUDIT scores in participants with BD and high DUDIT scores in SZ is in line with previous studies (7, 37), indicating some diagnostic preferences in the type of substance used. Even if the statistically significant differences in substance use between PNOS and the other diagnostic groups were largely based on the PNOS subgroup where SIPD could not be fully excluded, it is of interest that the PNOS group had high scores for both AUDIT and DUDIT, again placing it between SZ and BD.

Another frequent symptom constellation seen in our sample were the presence of persistent delusions with either overlapping affective episodes or impaired functioning. This symptom profile has significant overlaps with delusional disorder, which is a separate diagnostic entity characterized by delusions without the presence of prominent hallucinations, negative symptoms or functional loss (1, 10). In addition to having milder symptoms and better functioning, delusional disorder differs from SZ in that it occurs at a higher age (38) and from BD in that the delusions are present outside of mood episodes. Our findings here illustrate some of the limitations of categorical diagnostic systems, where somewhat narrow diagnostic criteria contribute to a possibly artificial distinction between specific psychotic disorders.

Along these lines, we also identified a group characterized by hallucinations (not in the form of commenting voices) without any other psychotic features. Hallucinations can be observed clinically in a wide range of psychiatric disorders outside of the psychotic spectrum, including post-traumatic stress disorder (39, 40), borderline personality disorder (41, 42) and anxiety disorder (43), and even in the general/non-psychiatric population. It has, therefore, been debated whether hallucinations alone should qualify for a psychotic disorder (44). Longitudinal studies of conditions presenting with hallucinations as the only psychotic feature are however still lacking.

The DSM-5, introduced in 2013, does not introduce any qualitative changes in the nosology of PPDs, including the syndromes captured by DSM-IV PNOS, but contains some modifications of diagnostic criteria for specific disorders. This could indirectly influence who will get a diagnosis of PNOS in ways that are difficult to predict. This might be the reason for dividing DSM-IV PNOS into a specific- and an unspecific diagnostic category, with the instruction to use the specified type where possible and stating the cause or type of symptomatology leading to the diagnosis (10, 45). This could be one way of tracking how changes in diagnostic criteria for a specific disorder influences its boundaries toward others. Another reason for the change is the decision to include APS into the DSM-5 Other specified schizophrenia spectrum disorders category. Individuals diagnosed with APS are not psychotic at the time of diagnosis, and their risk of developing psychosis is <25% (46). APS can thus not be labeled a psychotic disorder necessitating a change from “psychotic disorders” to “schizophrenia spectrum diagnosis” in the manual. Outside of this, the differences between DSM-IV PNOS and the new DSM-5 categories are minor.

DSM-5 explicitly places these two diagnostic categories within the schizophrenia spectrum. We, however, found a clear pattern of the PNOS group being consistently intermediate between SZ and BD when we examined for group differences for GAF and PANSS scores. The somewhat milder symptoms and better functioning of participants with PNOS may reflect that the basis for the PNOS diagnosis was either monosymptomatic delusions or monosymptomatic hallucinations, and less severe psychotic symptoms are also associated with better functioning (47, 48). However, a lower level of function could as well be expected due to the high prevalence of substance use in the PNOS group (49). The differences seen between participants with PNOS and SZ are consistent with findings from other studies (3, 16). One of the larger previous studies, including 904 patients with schizophrenia, schizoaffective or schizophreniform disorders and 150 patients with PNOS or brief psychosis, found more severe psychopathology and poorer functioning in the SZ group, but did not differentiate between PNOS and brief psychosis and did not include a BD group (3).

Since PNOS often is a provisional diagnosis, the development over time will be critical to our understanding of whether PNOS cases are “early stages” of specific disorders such as SZ or BD, or if it represents a group of stable syndromes falling outside of current criteria. While there are few cross-sectional studies of PNOS there are even fewer longitudinal studies specifically investigating the PNOS group and those that exist have mainly focused on diagnostic instability and risk of conversion to SZ or BD (50, 51). In adolescents diagnosed with PNOS, executive deficits and the absence of comorbid anxiety disorders have been identified as risk markers for SZ, while the presence of anxiety disorders have been associated with increased BD risk (50). A recent population-based study also identified the absence of anxiety- and other mood disorders, male gender, younger age and living in a low-income neighborhood as a risk factor for SZ in patients initially diagnosed as having PNOS (51). To understand the nature of the conditions classified as PNOS, we need more longitudinal studies also focusing on those that do not convert to BD or SZ.

### Strengths and Limitations

The strength of the present study is its large sample of participants diagnosed with PNOS through the use of SCID for DSM-IV by specially trained personnel with thorough evaluations of symptom profiles and -severity, as well as functioning and substance use.

However, the comprehensive assessment protocol may also have prohibited patients with very acute and unstable symptom presentations from participating, which may be the reason why we did not identify any participants with postpartum psychoses.

## Conclusions

To conclude, our study demonstrates that there is a need for a diagnostic category such as PNOS in categorically based diagnostic systems to capture the different psychotic syndromes not meeting the criteria for the specific diagnoses of SZ or BD. As expected, the symptom profiles captured by this diagnosis are heterogeneous and may have different needs when it comes to treatment. As a group, participants with PNOS are intermediate between participants with SZ and BD in terms of symptom severity and functional impairment. This indicates that many patients with PNOS will be in need of equally extensive and long-term treatment as patients with SZ and BD.

There is a need for longitudinal studies of patients initially diagnosed as PNOS, preferably with large sample sizes as the different symptom profiles may have different courses and outcomes.

## Data Availability Statement

The raw data supporting the conclusions of this article will be made available by the authors, without undue reservation.

## Ethics Statement

The studies involving human participants were reviewed and approved by Regional Committee for Medical and Health Research Ethics in Norway. The patients/participants provided their written informed consent to participate in this study.

## Author Contributions

LW and IM designed the study. LW performed the statistical analyses and supervised by IM. LW, IM, and CS wrote and edited the paper. CF, BH, RV, KW, GÅ, and TU have contributed with ideas to the study design and taken part in editing the paper. All authors contributed to the article and approved the submitted version.

## Conflict of Interest

The authors declare that the research was conducted in the absence of any commercial or financial relationships that could be construed as a potential conflict of interest.
